# The assessment of liver fibrosis in children with obesity on two methods: transient and two dimensional shear wave elastography

**DOI:** 10.1038/s41598-019-56358-2

**Published:** 2019-12-24

**Authors:** Cristina Oana Mărginean, Lorena Elena Meliţ, Dana Valentina Ghiga, Maria Oana Săsăran

**Affiliations:** 1 Department of Pediatrics, George Emil Palade University of Medicine, Pharmacy, Science, and Technology of Targu Mures, Gheorghe Marinescu street no 38, Targu Mures, 540136 Romania; 2 Department of Medical Informatics and Biostatistics, George Emil Palade University of Medicine, Pharmacy, Science, and Technology of Targu Mures, Gheorghe Marinescu street no 38, Targu Mures, 540136 Romania; 3 Department of Pediatric Cardiology, George Emil Palade University of Medicine, Pharmacy, Science, and Technology of Targu Mures, Gheorghe Marinescu street no 38, Targu Mures, 540136 Romania

**Keywords:** Non-alcoholic fatty liver disease, Paediatric research

## Abstract

The aim of this study was to assess the liver stiffness values in children with obesity versus healthy children on 2D-SWE and TE taking into account different laboratory parameters. We performed a case-control study on 287 children aged between 3 to 18 years, admitted in a Romanian Pediatric Tertiary Hospital, which we divided according to the body mass index (BMI) into two groups: the study group-77 children with obesity, and control group-210 children with normal weight. All children underwent anamnesis, clinical exam, laboratory parameters, ultrasound exam, and elastography. Children with obesity presented higher values of platelets, AST, ALT, and AAR as compared to control group (p = 0.0005/p = 0.0065/p < 0.0001/p < 0.0001). We found no significant differences for APRI between the two groups (p = 0.9827), although the values were higher in children with obesity. Significantly higher values of liver stiffness in children with obesity on both 2D-SWE and TE (p = 0.0314/p < 0.0001) were obtained. Similarly, the velocity values measured by 2D-SWE were also significantly higher in the study group (p < 0.0001). Our findings revealed significantly higher levels of platelets, transaminases, AAR, and liver stiffness values on both TE and 2D-SWE in children with obesity. 2D-SWE and TE might represent useful non-invasive methods for predicting liver impairment associated to pediatric obesity.

## Introduction

Childhood obesity has become a real health problem worldwide, and the World Health Organization (WHO) stated that over 40 million children are either overweight or obese^1^. The etiology of obesity is multifactorial, involving genetic and environmental factors^2–4^. A recent study performed on Romanian children, stated that 11.6% of the children above the age of 8 years suffer from obesity, and 26.8% are overweight^5^. Among the wide spectrum of obesity-related complications, nonalcoholic fatty liver disease (NAFLD) has become the most common hepatic chronic condition in developed countries, with an incidence between 5–15%^6^. The early inflammatory status associated to pediatric obesity is an important risk factor especially for long-term complications^7^. NAFLD might have a benign course, or on the contrary it might progress into nonalcoholic steatohepatitis (NASH) with further liver fibrosis, cirrhosis, neoplasia or organ failure^8^. The prevalence of NAFLD varies between 2.6–9.6% in overweight pediatric population^9^, and between 22.5 and 44% in children with obesity^9–11^. Even though hepatic biopsy is the gold-standard for the diagnosis of hepatic fibrosis, its usefulness in children is limited since it is an invasive method, and thus non-invasive methods for the assessment of liver fibrosis tend to be widely used^12,13^. Nevertheless, these non-invasive methods were studied especially on adults, while on pediatric population the data are rather scarce on this topic.

The two dimensional shear wave elastography (2D SWE) technique uses a region of interest (ROI) of approximately 20 cm^3^ based on a color display as homogeneous as possible. 2D-SWE is a novel, non-invasive method that assesses the elasticity of the liver tissue depending on the viscous and elastic properties of the hepatic tissue. It evaluates in real time the velocity (m/s) and stiffness (kPa), which are not influenced by respiratory phases, and it does not require for children to hold their breath during the examination^14^. The shear wave speed is measured in m/s and can be converted in elasticity/stiffness measured in kPa. During the examination the position of the color map must avoid great vessels^15^. In children, studies that aimed to assess this method are sporadic and included only a small number of healthy children, or with different chronic hepatic conditions, without establishing clear cutoff values in either healthy or non-healthy children^14,16,17^.

Transient elastography (TE) is another non-invasive and reproducible method for liver stiffness assessment in both children and adults. The TE device generates an elastic wave through a vibrator or special probe positioned on the thoracic wall, between the ribs, in order to be projected on the right liver lobe. After a correct positioning, this probe will transmit a low-amplitude signal to the hepatic tissue triggering an elastic shear wave that propagates through this tissue. Based on this process, TE allows the measurement of wave velocity, expressed in kPa, which is directly proportional to liver elasticity, varying from 2.5 to 74 kPa^6^.

It is a well-documented fact that certain laboratory parameters, especially liver transaminases are also useful in detecting liver impairment, though their levels are not enough for a proper assessment^18^. Thus, certain studies tried to identify different combinations between certain laboratory parameters which would have a higher accuracy in diagnosing liver fibrosis, among which aspartate aminotransferase (AST)/platelets ratio index (APRI)^19^, or AST/alanine aminotransferase (ALT) ratio (AAR)^20,21^.

***The aim*** of this study was assess the liver stiffness values in children with obesity versus healthy children on 2D-SWE and TE taking into account different laboratory parameters.

## Results

### Descriptive analysis

In our study, the mean age for the study group was 10.44 ± 3.38 years, while in the control group it was 11.29 ± 3.83 years, without statistical significance (p = 0.0745), thus we can say that our groups were similar in terms of age distribution. Regarding gender distribution, we noticed a predominance of females (54.76%) in control group as compared to the study group where we encountered a higher proportion of males, 66.23% versus 33.77% females. Our findings revealed that obesity was more frequent among boys (p = 0.0016).

Regarding the laboratory parameters, children with obesity were found to have significantly higher values of platelets, 336.40 ± 90.12 × 10^3^/µl as compared to the normal weight ones, 298.50 ± 67.27 × 10^3^/µl (p = 0.0005). Similarly, the values of AST and ALT, as well as the AAR were significantly higher in children with obesity in comparison to control group (p = 0.0065/p < 0.0001/p < 0.0001). Contrariwise, we found no significant differences in terms of APRI between the two groups (p = 0.9827), although the values were higher in children with obesity, 0.22 ± 0.20 as compared to those encountered in control group, 0.20 ± 0.12.

As we mentioned, all children were initially assessed by 2D abdominal ultrasound. Based on this examination, we encountered the following pathological changes in children with obesity: 24 (31.17%) presented hepatomegaly, 41 (53.25%) were encountered with both hepatomegaly and sings of hepatic steatosis, while 12 patients (15.58%) were found only with signs of liver steatosis.

Liver elastography studies revealed significantly higher values of liver stiffness in children with obesity on both 2D-SWE (3.84 ± 0.35 kPa in the study group versus 3.73 ± 0.48 kPa in control group, p = 0.0314) and TE (4.23 ± 0.53 kPa in children with obesity versus 3.80 ± 0.48 kPa in control group, p < 0.0001). Similarly, the velocity values measured by 2D-SWE were also significantly higher in the study group, 1.18 ± 0.09 m/s versus 1.09 ± 0.09 m/s in control group (p < 0.0001).

The values of the parameters mentioned above are described in Table 1.

**Table 1:** The descriptive analysis of transaminases, APRI, AST/ALT index and elastography parameters for our sample.

Parameters	Study group (n = 77) Mean ± SD (Median)	Control group (n = 210) Mean ± SD (Median)	p value
Age (years)	10.44 ± 3.38 (11.00)	11.29 ± 3.83 (11.00)	*0.0745
Platelets (x10^3^/µl)	336.40 ± 90.12 (321.0)	298.50 ± 67.27 (289.0)	*0.0005
AST (UI)	27.33 ± 23.62 (21.90)	22.29 ± 10.96 (20.60)	*0.0065
ALT (UI)	26.50 ± 43.13 (18.10)	13.64 ± 6.96 (12.40)	*<0.0001
APRI	0.22 ± 0.20 (0.17)	0.20 ± 0.12 (0.1702)	*0.9827
AST/ ALT	1.37 ± 0.57 (1.31)	1.74 ± 0.67 (1.628)	*<0.0001
2D-SWE (kPa)	3.84 ± 0.35 (3.79)	3.73 ± 0.48 (3.72)	**0.0314**
TE (kPa)	4.23 ± 0.53 (4.15)	3.80 ± 0.48 (3.89)	*<0.0001
V median (m/s)	1.18 ± 0.09 (1.160)	1.09 ± 0.09 (1.09)	*<0.0001

ALT – alanine aminotransferase, AST – aspartate aminotransferase, CI – confidence interval, 2D-SWE – 2D-Shear Wave Elastography, TE – transient elastography, E – elasticity, kPa – Kilo Pascal, m/s – meter/second, n – number, SD – standard deviation, V – velocity * Mann-Whitney test was used.

### Correlations between laboratory, liver stiffness and velocity parameters

Analyzing the correlations between the mean values of liver stiffness (E median) obtained on 2D-SWE and laboratory parameters, we obtained a positive correlation (direct dependency) between E median and platelets count (r = 0.06973; 95% CI: [−0.0498 to 0.1874]), as well as between E median and ALT (r = 0.07280; 95% CI: [−0.0468 to 0.1904]), but without statistical significance (p = 0.2389/p = 0.2189). Contrariwise, we noticed a reverse correlation between E median and both AST (r = −0.008045; 95% CI: [−0.1271 to 0.1113]), and APRI (r = −0.04567; 95% CI: [−0.1640 to 0.07393]), as well AAR index (r = −0.04661; 95% CI: 0.1649 to 0.07300]), but also without statistical significance (p = 0.8921/p = 0.4408/p = 0.4315) (Table 2).

**Table 2:** Correlations between laboratory parameters and elastography parameters on both 2D-SWE and TE.

Parameters	E Median 2D-SWE (kPa)			E Median TE (kPa)			V median (m/s)		
	r coefficient	95% CI	p value	r coefficient	95% CI	p value	r coefficient	95% CI	p value
**Platelets** (x10^3^/µl)	0.06973	−0.0498 to 0.1874	0.2389	0.1140	−0.005309 to 0.2300	0.0538	0.09945	−0.01999 to 0.2161	0.0926
**AST** (UI)	−0.008045	−0.1271 to 0.1113	0.8921	−0.04373	−0.1621 to 0.07587	0.4606	0.07880	−0.04079 to 0.1962	0.1831
**ALT** (UI)	0.07280	−0.0468 to 0.1904	0.2189	0.07462	−0.04498 to 0.1921	0.2075	0.2375	0.1217 to 0.3469	**<0.0001**
**APRI**	−0.04567	−0.1640 to 0.07393	0.4408	−0.1084	−0.2247 to 0.01096	0.0667	−0.006338	−0.1254 to 0.1129	0.9149
**AAR**	−0.04661	−0.1649 to 0.07300	0.4315	−0.1215	−0.2372 to −0.002294	**0.0398**	−0.2100	−0.3212 to −0.09315	**0.0003**

ALT – alanine aminotransferase, AST – aspartate aminotransferase, CI – confidence interval, 2D-SWE – 2D-Shear Wave Elastography, TE – transient elastography, E – elasticity, kPa – Kilo Pascal, m/s – meter/second, V – velocity.

On the other hand, when assessing liver stiffness by TE, we encountered a positive correlation (direct dependency) between E median and platelets count (r = 0.1140; 95% CI: [−0.005309 to 0.2300]), but also ALT (r = 0.07462; 95% CI: [−0.04498 to 0.1921]), also with no statistical significance (p = 0.0538/p = 0.2375). Moreover, similar to the results obtained by 2D-SWE, we noticed also a reverse correlation between E median on TE and both AST (r = −0.04373; 95% CI: −0.1621 to 0.07587]), and APRI (r = −0.1084; 95% CI: [−0.2247 to 0.01096]), both without statistical significance (p = 0.4606/p = 0.0667). Additionally we found a significant negative correlation between E Median on TE and AAR index (r = −0.1215; 95% CI: [−0.2372 to −0.002294]), p = 0.0398 (Table 2).

In terms of velocity (V Median), we identified positive correlations with platelets count (r = 0.09945; 95% CI: [−0.01999 to 0.2161]), and AST (r = 0.07880; 95% CI: −0.04079 to 0.1962), both without statistical significance (p = 0.0926/0.1831). Moreover, we found a significant positive correlation between V Median and ALT (r = 0.2375, 95% CI: [0.1217 to 0.3469], p < 0.0001). On the other hand, V Median was found to be negatively correlated with both APRI (r = −0.006338; 95% CI: [−0.1254 to 0.1129]) and AAR (r = −0.2100; 95% CI: [−0.3212 to −0.09315]), but significant correlation was noticed only in case of AAR (p = 0.0003) (Table 2).

It is a well-documented fact that liver ultrasound aspect differs depending on age, and thus, we divided our study sample into 4 age groups: 3–5 years, 6–9 years, 10–13 and 14–18 years of age. Of the entire sample, 14 normal weight children (6.67%) and 9 children with obesity (11.69) belonged to the age group 3–5 years; in the next age group, 6–9 years, we had 57 (27.14%) children from the control group versus 20 children (25.07%) in the study group; 64 children (30.48%) from the control group and 34 children (44.16%) from the group with obesity were between 10–13 years of age; while, in the last age group, 14–18 years, we found 75 normal weight children (35.71%), versus 14 children with obesity (18.18%). In terms of BMI distribution on the same age groups, we identified a mean BMI of 18.27 ± 3.41 kg/m^2^ in control group versus 24.67 ± 8.16 kg/m^2^ in the study group for the age group 3–5 years; 17.86 ± 3.17 kg/m^2^ in normal weight children between 6–9 years of age and 24.86 ± 3.38 kg/m^2^ for obese children with the same age range; for the age group 10–13 years, the mean BMI was 19.33 ± 3.08 kg/m^2^ in normal weight group and 29.17 ± 4.94 kg/m^2^ in obese group; whereas in the last age group, 14–18 years, we encountered a mean BMI of 19.67 ± 2.81 kg/m^2^ in control group and 31.43 ± 3.16 kg/m^2^ in the study group (Table 3).

**Table 3:** The distribution of our population on age groups and BMI.

Parameters	BMI (kg/m^2^)	
Age	Study group (n = 77) Mean ± SD (Median)	Control group (n = 210) Mean ± SD (Median)
Total group	27.14 ± 5.15 (26.20)	18.98 ± 3.10 (18.50)
3–5 years	24.67 ± 8.16 (22.50)	18.27 ± 3.41 (17.65)
6–9 years	24.86 ± 3.38 (24.00)	17.86 ± 3.17 (17.40)
10–13 years	29.17 ± 4.94 (28.30)	19.33 ± 3.08 (18.50)
14–18 years	31.43 ± 3.16 (30.95)	19.67 ± 2.81 (19.60)

BMI – body mass index; n – number, SD – standard deviation,*Mann-Whitney test was used.

The value of 2D-SWE, TE and V median in the two groups, according to the age groups is described in Table 4. Thus, the values of E Median by 2D-SWE were: in the age group 3–5 years 3.98 ± 0.37 KPa for children with obesity, significantly higher as compared to those in control group, 3.60 ± 0.27 kPa (p = 0.018); in the age group 6–9 years, 3.78 ± 0.34 kPa in the study group versus 3.76 ± 0.41 kPa in normal weight children (p = 0.9075); for children with the age between 10–13 years, we found a mean value of 3.91 ± 0.33 kPa in the study group versus 3.75 ± 0.48 kPa in control group (p = 0.0838); whereas for the last age group, 14–18 years, we identified 3.78 ± 0.47 kPa in children with obesity versus 3.78 ± 0.47 kPa in control group (p = 0.9705). The values of E Median recorded by TE in the two groups (Table 4): 4.04 ± 0.30 kPa in the study group versus 3.66 ± 0.37 KPa in control group for the age group 3–5 year (p = 0.0412); in the age group 6–9 years, 4.10 ± 0.53 kPa in children with obesity as compared to 3.65 ± 0.42 KPa for normal weight children (p = 0.0032); for children with the age between 10–13 years, 4.28 ± 0.52 kPa in case of those with obesity versus 3.80 ± 0.45 kPa in control group (p = 0.0004); while for the last age group, 14–18 years, 4.57 ± 0.55 kPa in case of children with obesity and 3.96 ± 0.53 kPa in normal weight ones (p = 0.0013). The distribution of mean values of V Median by 2D-SWE according to the 4 age groups were (Table 4): in children between 3–5 years, 1.19 ± 0.07 m/s in the study group versus 1.06 ± 0.08 m/s in control group (p = 0.0034); 6–9 years age group, 1.20 ± 0.09 m/s in children with obesity as compared to 1.10 ± 0.05 m/s in normal weight ones (p < 0.0001); 10–13 years age group, 1.17 ± 0.09 m/s in the group with obesity in comparison to 1.08 ± 0.13 m/s in normal weight group (p < 0.0001); while for children with the age between 14–18 years, 1.15 ± 0.07 m/s among children with obesity versus 1.10 ± 0.07 m/s in control group (p = 0.0140). Thus, all values of both E Median by TE and V Median by 2D-SWE were significantly higher in children with obesity in comparison to normal weight ones for all age groups.

**Table 4:** 2D-SWE, TE and V median according the age.

Parameters	Age	Study group (n = 77) Mean ± SD (Median)	Control group (n = 210) Mean ± SD (Median)	p value
**2D-SWE (kPa)**	3–5 years	3.98 ± 0.37 (3.880)	3.60 ± 0.27 (3.575)	**0.0187**
	6–9 years	3.78 ± 0.34 (3.760)	3.76 ± 0.41 (3.720)	0.9075
	10–13 years	3.91 ± 0.33 (3.945)	3.75 ± 0.48 (3.750)	0.0839
	14–18 years	3.78 ± 0.47 (3.665)	3.71 ± 0.55 (3.750)	*0.9705
**TE (kPa)**	3–5 years	4.04 ± 0.30 (3.990)	3.66 ± 0.37 (3.745)	**0.0412**
	6–9 years	4.10 ± 0.53 (4.210)	3.65 ± 0.42 (3.700)	**0.0032**
	10–13 years	4.28 ± 0.52 (4.075)	3.80 ± 0.45 (3.800)	*0.0004
	14–18 years	4.57 ± 0.55(4.620)	3.96 ± 0.53 (3.910)	*0.0013
**V median (m/s)**	3–5 years	1.19 ± 0.07 (1.160)	1.06 ± 0.08 (1.085)	**0.0034**
	6–9 years	1.20 ± 0.09 (1.150)	1.10 ± 0.05 (1.100)	*<0.0001
	10–13 years	1.17 ± 0.09 (1.160)	1.08 ± 0.13 (1.080)	*<0.0001
	14–18 years	1.15 ± 0.07 (1.140)	1.10 ± 0.07 (1.080)	*0.0140

n – number, SD – standard deviation, 2D-SWE – 2D-Shear Wave Elastography, TE – transient elastography, E – elasticity, kPa – Kilo Pascal, m/s – meter/second, V – velocity, *Mann-Whitney test was used.

Elastography parameters (E Median and V Median) determined by both 2D-SWE and E Median on TE were assessed using the area under the receiver operating characteristic (ROC) curves.

**Figure 1:**
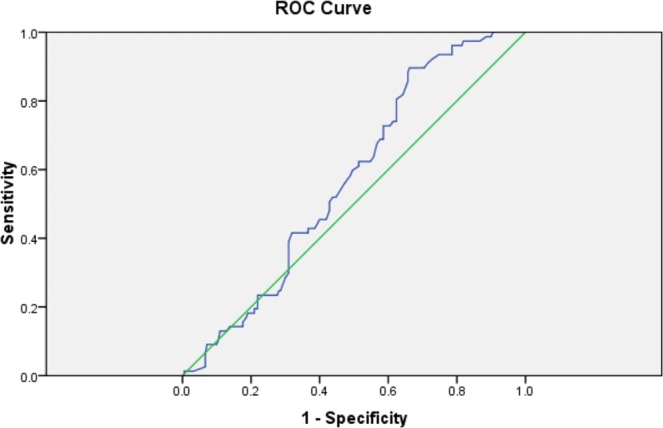
ROC curve of E Median determined by 2D-SWE in prediction of the liver fibrosis. 2D-SWE- two-dimensional shear wave elastography, E- elasticity, ROC curve- receiver operating characteristic curve.

ROC curves of E median values (kPa) determined on 2D-SWE for discriminating liver stiffness were plotted in the Fig. 1. The AUC was 0.573 ± 0.035 [CI 95%: 0.505–0.642] (p = 0.057). Thus, the assessment of E Median in 2D-SWE might not be enough for an accurate differentiation between children with or without liver impairment (steatosis or fibrosis) associated to obesity.

**Figure 2:**
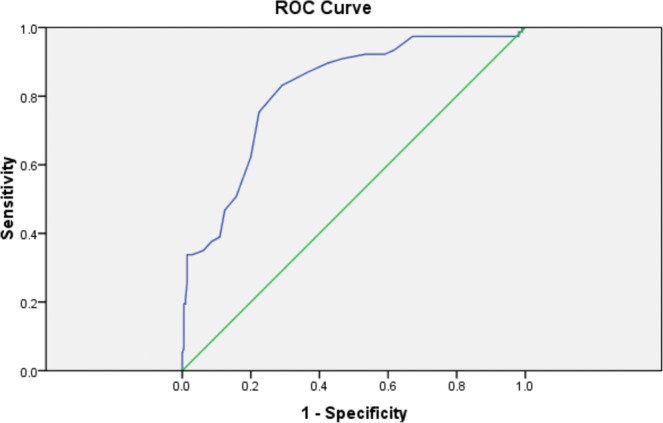
ROC curve of V median in prediction of the liver fibrosis. V- velocity, ROC curve- receiver operating characteristic curve.

ROC curves of V median (m/s) values for discrimination of liver velocity were plotted in the Fig. 2. The AUC was 0.817 ± 0.028 [CI 95%: 0.762–0.872] (p = 0.0000). Thus, due to its high accuracy encountered in our study, velocity might be an important indicator of obesity-associated liver impairment.

**Figure 3:**
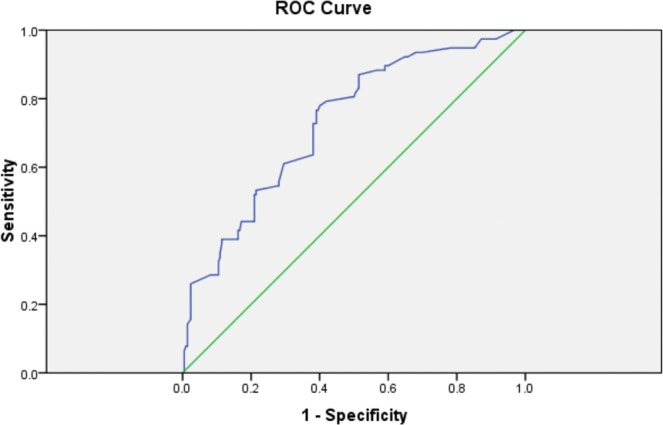
ROC curve of E Median determined by TE in prediction of the liver fibrosis. E: elasticity, ROC curve: receiver operating characteristic curve, TE: transient elastography.

ROC curves of E median (kPa) determined on TE in order to discriminate liver stiffness were plotted in the Fig. 3. The AUC was 0.730 ± 0.033 [CI 95%: 0.665–0.794] (p = 0.0000). Our findings showed that this test has a high accuracy, thus E Median on TE might represent a useful tool in differentiating between liver impairment associated to obesity and normal liver tissue in children.

We also performed a post-hoc analysis, which revealed that the power of the study was: 58.5% for E Median by 2D-SWE (kPa), 100% for E Median by TE (kPa), and 100% for V Median by 2D-SWE (m/s). Thus, we might state that in our sample, liver stiffness assessed by TE and velocity by 2D-SWE are better predictors as compared to liver stiffness of liver impairment due to obesity.

## Discussions

Elastography is a useful diagnostic tool for liver steatosis or fibrosis associated to different pathologies. Both 2D-SWE and TE were widely used in adults, but unfortunately, the data in pediatric population is scarce resulting in a lack of accurate reference values for liver stiffness in both healthy children and those with different conditions that might lead to liver fibrosis. Pediatric obesity, a real health problem worldwide is well-known to lead to liver fibrosis as a long-term complication. A very recent study performed on pediatric patients with different degrees of obesity proved that liver stiffness increases with obesity severity underlining that both liver steatosis and fibrosis might be assessed by non-invasive elastography methods^22^.

Studies performed on obese children are controversial. Thus, certain studies performed on children with obesity and NASH showed that 2D-SWE combined with simple laboratory tests for the assessment of hepatic function provided accurate parameters for the quantification of liver fibrosis^12,23^. Similarly, Yoneda *et al*. established a positive correlation between median velocity measured by acoustic force impulse radiation (ARFI) and liver fibrosis in patients with NAFLD^24^. Another study of our team that assessed liver fibrosis on ARFI technology in children with malignant conditions, non-malignant liver disease, children with obesity and overweight, but also healthy controls, pointed out that the group with NAFLD expressed the highest values of elastography parameters^25^. Also, the recent review of Sleman *et al*. underlined the importance of ultrasound elastography as a method for assessing the evolution and prognosis of chronic liver conditions in children^26^. Bailey *et al*. also emphasized the important role of 2D-SWE as a quantitative biomarker for the assessment of liver stiffness in children with obesity^18^. The authors encountered significantly higher values of SWE velocity in children with obesity, 1.44 ± 0.39 m/s versus 1.08 ± 0.14 m/s in normal weight ones, suggesting that obesity results in stiffer livers (p < 0.001)^18^. Our findings also pointed out significantly higher values of velocity on 2D-SWE in the study group, 1.18 ± 0.09 m/s versus 1.09 ± 0.09 m/s in control group (p < 0.0001). The velocity value obtained in our control group was almost identical as compared to that noticed by Bailey *et al*., while the one encountered in children with obesity was higher in case of the latter study, 1.44 ± 0.39 m/s versus 1.18 ± 0.09 m/s in our group of children with obesity. This difference might be explained by the higher number of cases with obesity, the different device used for elastography measurements, and probably the higher absolute values of BMI of children with obesity included in the study performed by Bailey *et al*.^18^. Contrariwise, the study of Ozkan *et al*. pointed out limitations of this methods regarding the differentiation between low-grade fibrosis and normal liver tissue^27^. Nevertheless, since 2D-SWE is the most recent technique among all elastography techniques, the controversies regarding its usefulness in patients with NAFLD result especially from the small number of studies reported in the literature.

Transient elastography (Fibroscan) proved its utility in adult population, but also in children, in whom the few studies reported in the literature proved that it is a useful, fast and reproducible method for the assessment of liver fibrosis^14,17,28^ in different chronic disorders^6,29^. Moreover, Cho *et al*. in a study performed on 201 children established the usefulness of TE as a non-invasive method for the screening of steatohepatitis and hepatic fibrosis in Japanese children with obesity^30^. The pediatric population included in this study was divided into three groups: children with obesity, children with liver disease, and control groups. The authors found significantly higher values of liver stiffness in children with obesity, 5.5 ± 2.3 kPa in comparison to normal weight children and liver disease group, 3.9 ± 0.9 kPa (p < 0.001), as did our study. Only 4 patients with obesity and 1 patient with type B hepatitis and obesity included in the previously mentioned study underwent liver biopsy, but the results showed that TE parameters were highly correlated with both fibrosis and steatosis degree found in the histological exam. Nevertheless, several studies stated that SWE is more accurate for the assessment of liver fibrosis as compared with TE and ARFI, especially in patients with chronic viral hepatitis^31–33^. Our study assessed both methods, 2D-SWE and TE for the assessment of liver stiffness in children with obesity, and our findings pointed out that both velocity values obtained on 2D-SWE and liver stiffness ones on TE are accurate indicators of liver fibrosis associated to pediatric obesity. Moreover, we found the stiffness values on both techniques to be significantly higher among children with obesity as compared to normal weight ones. Thus, we can state that both methods might be confidently used for the non-invasive assessment of liver fibrosis associated to obesity in pediatric patients.

Regarding laboratory parameters, the most constant finding consists in higher values of liver transaminases encountered in patients with obesity^34^, but this single aspect is not enough for the precise identification of hepatic fibrosis degree^20^. Thus, is spite of the well-documented higher levels of AST and ALT in pediatric patients with obesity, only ALT seems to be positively correlated with liver stiffness measured by TE^30^. Our study also proved positive correlations between all elastography parameters and ALT levels, but statistical significance was noticed only in case of velocity values. Despite the fact that patients with chronic liver disease are usually found with thrombocytopenia, our study proved that children with obesity express an increased level of platelets. Multiple factors might explain this finding, among which: the normal spleen sizes, the normal function of the bone marrow revealed by CBC parameters^35^, the early inflammatory status-associated to pediatric obesity^7^, or even their involvement in the healing of acute liver injuries^36^ caused by obesity, thrombocytosis being a possible compensatory mechanisms in these patients.

Thus, early stages of obesity encountered in children might associate an increased platelets count as a compensatory mechanism designed to heal acute liver injuries related to this condition and even to prevent the development of liver fibrosis.

Nevertheless, multiple studies focused on combining different laboratory parameters and assessing their usefulness in predicting liver fibrosis. Thus, Nobili *et al*. proved a significant positive correlation between hepatic fibrosis degree and APRI^19^. Cho *et al*. also proved that childhood and adolescent obesity are associated with higher levels of APRI, but they failed in finding a positive correlation between this index and liver stiffness values^30^. Our study also revealed higher values of APRI in children with obesity as compared to control group, and underlined a reverse correlation between this index and all elastography parameters. Moreover, a value of AAR above 1 was associated with hepatic fibrosis, and even cirrhosis^20^. Nevertheless, studies proved that this marker has a sensitivity of only 50% for cirrhosis prediction, and an even lower sensitivity for advanced degrees of fibrosis^20^. Contrariwise, our study pointed out significantly higher levels of AAR in children with obesity, but failed in identifying a positive correlation between this indirect marker and the elastography parameters measured by both techniques.

A multidisciplinary approach involving physicians and dietetics specialists with distinguished communication skills is mandatory in order to elaborate strategies to decrease the incidence of pediatric obesity and to properly monitor their effectiveness^37^. The implementation of national screening programs similar to other pathologies^38,39^ using elastography methods for the assessment of liver fibrosis associated to pediatric obesity would be really useful regarding the long-term prognosis of these patients.

The limitations of our study consist mainly in the relatively small number of children with obesity, but also the fact that we did not assess children below the age of 3 years. Even though, this might also represent a strength due to the lack of compliance in small children that could result in assessment failure or false measurements. Moreover, liver biopsy would have been useful for a more accurate correlation, but its indications in children are limited, and moreover, it has no indication in healthy controls. On the other hand, our study has multiple strengths: the size of the control group that provides an increased statistical power, the fact that we assessed liver stiffness on two elastography methods combined with different laboratory parameters, the high number of determinations for each patient and each parameter, but also the accuracy of the statistical analysis.

To the best of our knowledge this is the first study in Romania, and probably among the few worldwide that assessed liver fibrosis associated to pediatric obesity using two elastography methods combined with other laboratory parameters.

## Conclusions

Elastography is a valuable non-invasive method that should be used in order to assess liver fibrosis associated to obesity in children since pediatric obesity reached alarming rates worldwide during the last decades. Our findings revealed significantly higher levels of platelets, transaminases and AAR in children with obesity as compared to normal weight ones. Moreover, liver stiffness values assessed on both TE and 2D-SWE were significantly higher in the same group. Liver stiffness values on both methods were positively correlated with platelets count and ALT values, but negatively correlated with AST, APRI and AAR. Assessing the specificity and sensitivity of the elastography methods used in our study, our study suggests that 2D-SWE and TE might represent useful non-invasive methods for predicting liver impairment associated to pediatric obesity. Nevertheless, further studies are needed on larger samples in order to identify the precise reference values of liver fibrosis in children with obesity, taking into account if possible, the confirmation of these values through liver biopsy.

## Methods

### Ethics approval and informed consent

All parents/caregivers granted their consent by signing the informed consent prior to their children inclusion in the study. Moreover, the study was explained to all children in order to obtain their assent for participating in our study. The Ethics Committee of the University of Medicine and Pharmacy of Târgu Mureș approved our study (No 329/ November 17^th^ 2017), and it was performed according to the principles of the Helsinki Declaration.

### Study sample selection

We performed a cross-sectional study on 287 children aged between 3 to 18 years, admitted in a Romanian Pediatric Tertiary Hospital, from September 2017 to July 2019. The study sample was divided according to the body mass index (BMI) into two groups: group 1, the study group, comprising 77 children with obesity (BMI Percentile (P) ≥95), and group 2, control group, comprising 210 children with normal weight (BMI P ≥ 5 and <85). The inclusion criteria for the study group consisted in children with BMI above P_95_, age between 3 and 18 years, without any history of acute, chronic or genetic conditions; while for control group: clinically healthy children, BMI P ≥ 5 and <85, age between 3 and 18 years, no history of any chronic or acute pathology, normal laboratory parameters, normal morphology and echogenicity of the liver parenchyma, who were usually brought for a routine medical consult as a result of their parents/caregivers choice. The exclusion criteria for both groups consisted in age below 3 years or above 18 years, history of acute or chronic pathologies, diagnosis of genetic syndromes or chromosomal anomalies, but also children whose parents refused to sign the informed consent form prior to the inclusion in the study. All children were assessed on a one-day chart system and they did not require longer hospitalization.

### Variables of interest

Initially, both children with obesity and normal weight ones underwent a thorough anamnesis and clinical exam. Only those who fulfilled the inclusion and exclusion criteria after this first step, were submitted to the next one, the assessment of different laboratory parameters, among which complete cellular blood count (CBC) in order to obtain the platelets count, C-reactive protein for the exclusion of an acute, and liver transaminases (AST and ALT), AAR and APRI.

The laboratory parameters were assessed using a Cobas Integra 400 plus automated analyzer (Roche Diagnostics GmbH, Mannheim, Germany).

Ultrasound exam started with an assessment of abdominal organs, and especially liver morphology and echogenicity in all children. The right liver lobe was assessed on the anterior axillary line by simultaneous visualization of the right kidney. Liver sizes were interpreted according to the normal values for age and gender^40^. Bright liver echo pattern was interpreted as an ultrasonography sign of liver steatosis. Afterwards, all children were examined by both TE and 2D-SWE, benefiting from 12 measurements for each method (12 for liver stiffness on 2D-SWE, 12 for velocity on 2D-SWE, and 12 for liver stiffness on TE), and only their median was considered in our study for a better accuracy of the examination, taking into account an interquartile range interval (IQR) ≤ 30% for each patient.

The results were interpreted in m/s and kPa for 2D-SWE and kPa for TE.

In case of 2D-SWE, all measurements were performed with a Logiq S8 General Electrics Machine (General Electric Healthcare, Wauwatosa, WI, USA), using a C1-6 convex probe, at approximately 2 cm under the Glisson’s capsule, on a color map with over 50% homogeneity. FibroScan device General Electrics (EchoSens, Paris, France) was used for TE assessment, using M or XL probe according to BMI.

All children were examined after a fasting period of approximately 6 hours, each exam lasted approximately 20 minutes, without sedation and all measurements were performed by a highly experienced physician with over 10 years’ experience in pediatric ultrasound, and 3 years in elastography.

### Statistical analysis

The statistical analysis included descriptive elements (frequency, mean, standard deviation, confidence interval 95%) and elements of inferential statistics. The Shapiro-Wilk test was applied in order to assess the distribution of analyzed series of data. For the comparison of means, we used t-Student test for unpaired data with Welch and Mann-Whitney test, non-parametric test for median comparison. Chi squared test and Spearman tests were used in order to identify the associations and correlations between the qualitative variables. We also assessed the area under the receiver operating characteristic curve **-** ROC (AUC – area under the curve) in order to determine the accuracy of the elastography methods. We applied one sample t test to compare the mean of sample data to a known value and we used Bland Altman graphic as a method to represent the data. The significance threshold for p value was 0.05. The statistical analysis was performed based on GraphPad Prism trial variant software and the SPSS software.

**What is known:**

- Nonalcoholic fatty liver disease (NAFLD) might have a benign course, or on the contrary it might progress into nonalcoholic steatohepatitis (NASH) with further liver fibrosis, cirrhosis, neoplasia or organ failure
- 2D-SWE is a novel, non-invasive method that assesses the elasticity of the liver tissue depending on the viscous and elastic properties of the hepatic tissue.
- Transient elastography (TE) is another non-invasive and reproducible method for liver stiffness assessment in both children and adults.

**What is new:**

- Our study revealed significantly higher levels of platelets, transaminases and AAR in children with obesity as compared to normal weight one. Liver stiffness values on both methods were positively correlated with platelets count and ALT values, but negatively correlated with AST, APRI and AAR.
- Based on our findings, 2D-SWE and TE might be considered useful non-invasive indicators of liver fibrosis associated to pediatric obesity.
